# *Cryptosporidium* as a testbed for single cell genome characterization of unicellular eukaryotes

**DOI:** 10.1186/s12864-016-2815-y

**Published:** 2016-06-23

**Authors:** Karin Troell, Björn Hallström, Anna-Maria Divne, Cecilia Alsmark, Romanico Arrighi, Mikael Huss, Jessica Beser, Stefan Bertilsson

**Affiliations:** Department of Microbiology, National Veterinary Institute, Uppsala, Sweden; Department of Microbiology, Public Health Agency of Sweden, Solna, Sweden; Microbial Single Cell Genomics Facility, Department of Cell and Molecular Biology and Science for Life Laboratory, Uppsala University, Uppsala, Sweden; Division of Pharmacognosy, Department of Medicinal Chemistry, Biomedical Center, Uppsala University, Uppsala, Sweden; Department of Biochemistry and Biophysics, Science for Life Laboratory, Stockholm University, Solna, Sweden; Department of Ecology and Genetics, Limnology and Science for Life Laboratory, Uppsala University, Uppsala, Sweden

**Keywords:** Apicomplexa, Single cell genomics, Whole genome amplification, *Cryptosporidium*, Multiple infection, FACS

## Abstract

**Background:**

Infectious disease involving multiple genetically distinct populations of pathogens is frequently concurrent, but difficult to detect or describe with current routine methodology. *Cryptosporidium* sp. is a widespread gastrointestinal protozoan of global significance in both animals and humans.

It cannot be easily maintained in culture and infections of multiple strains have been reported.

To explore the potential use of single cell genomics methodology for revealing genome-level variation in clinical samples from *Cryptosporidium*-infected hosts, we sorted individual oocysts for subsequent genome amplification and full-genome sequencing.

**Results:**

Cells were identified with fluorescent antibodies with an 80 % success rate for the entire single cell genomics workflow, demonstrating that the methodology can be applied directly to purified fecal samples. Ten amplified genomes from sorted single cells were selected for genome sequencing and compared both to the original population and a reference genome in order to evaluate the accuracy and performance of the method. Single cell genome coverage was on average 81 % even with a moderate sequencing effort and by combining the 10 single cell genomes, the full genome was accounted for. By a comparison to the original sample, biological variation could be distinguished and separated from noise introduced in the amplification.

**Conclusions:**

As a proof of principle, we have demonstrated the power of applying single cell genomics to dissect infectious disease caused by closely related parasite species or subtypes. The workflow can easily be expanded and adapted to target other protozoans, and potential applications include mapping genome-encoded traits, virulence, pathogenicity, host specificity and resistance at the level of cells as truly meaningful biological units.

**Electronic supplementary material:**

The online version of this article (doi:10.1186/s12864-016-2815-y) contains supplementary material, which is available to authorized users.

## Background

The genus *Cryptosporidium* belongs to the phylum Apicomplexa, which comprises many parasites of medical and veterinary importance, including *Plasmodium, Toxoplasma, Sarcocystis* and *Eimeria*. In developing countries, cryptosporidiosis is very common in children and has recently been identified as one of the leading causes of childhood diarrhoeal disease [1]. *Cryptosporidium* sp. can infect both humans and other animals, and different species have different pathogenicity and host specificity. There are 26 species described to date and the number of newly named species is increasing continuously [2]. Of the nearly 20 species and genotypes described in humans [2], some species are host specific while others have a broader host range, such as the zoonotic *C. parvum* and *C. ubiquitum*. Thus, molecular characterization of *Cryptosporidium* sp. has high epidemiological relevance both in surveillance, outbreak investigations and for studies of parasite biology. *Cryptosporidium* is spread by infective, sporulated oocysts. Each oocyst contain four sporozoites, each with a haploid genome. The oocyst, which is the form exiting the host through feces is a dormant stage, ready to infect its next host. After ingestion by a host the oocyst releases the sporozoites which invade the intestinal epithelial cells. The parasite undergo asexual reproduction and later a sexual reproductive stage. The result, an oocyst, is passed through feces and hence the only external life form (as well as post meiosis) and is therefore a suitable target for detection and further genomic studies.

For identification of *Cryptosporidium* isolates, amplification of the 18S rRNA and restriction fragment length polymorphism (RFLP) and/or sequencing is commonly used [2]. Subtyping can be performed within each species and at least for the most important species infectious to humans, the gp60 gene is used for this purpose [2–5]. It is known from several studies that multiple infections accrue, both with several species infecting the same host [6, 7], but also with several gp60 subtypes of *C. parvum* detected in one single isolate [8]. Hence the epidemiology of *Cryptosporidium* outbreaks and sporadic cases, especially from endemic regions, can be complex and require differentiation of mixed populations.

Aside from very promising work published by Morada et al. [9] there is no established method for continuous culture of *Cryptosporidium*, and the use of animals to passage and amplify the parasites was performed to propagate sufficient material for genome sequencing. However, there have recently been some advances in the isolation and whole genome sequencing of *Cryptosporidium* from clinical samples [3, 10–12]. The genome sequences from clinical isolates available today have been obtained in procedures involving a step of immunomagnetic separation (IMS) and are limited to samples with relatively high parasite burden (≥10^3^ oocysts per gram, OPG). Such genomes are derived from combined communities that apart from other non-target organisms, may host multiple genetically distinct variants and thus represents a complex metagenome.

In contrast to metagenomic approaches, the emerging field of single cell genomics has, for the first time, enabled researchers to acquire and analyze genomic data from individual cells of interest, including those that cannot as of yet be cultured [13–15]. The workflow involves initial single cell partitioning followed by lysis and whole genome amplification prior to downstream genome sequencing [16]. Single cell genome sequencing is a reliable way to robustly examine and describe cellular level genetic variation in complex populations, particularly low frequency variation. Using other methods, this potentially great microdiversity may be masked, overlooked and thus lost [13, 17].

The isolation of individual cells for single cell genome sequencing is often performed on fluorescence activated cell sorting (FACS) platforms [18–20], but other approaches, such as microfluidic devices, microdroplets and laser tweezers also hold promise [17, 21]. There are many potential applications of this methodology that could be of relevance from a public health perspective [15, 21, 22], but the use in parasitology is so far largely unexplored. Recently, Nair et al. [23] for the first time published a study describing successful isolation, whole genome amplification and genome sequencing of eukaryote parasites in individual blood cells. Each blood cell supposedly contains one to four malaria parasite genome copies [23] and hence this study clearly demonstrates the promise, but also the challenges in adopting existing single cell genomics workflows to study the biology and diversity of this type of medically important microorganisms. Still, the great diversity in protozoa, calls for additional adaptation and validation of the methodology to account for contrasting genome features, susceptibility to isolation, lysis and DNA extraction. Some progress in such broader attempts to apply single cell genomics to protozoa has been reported for biodiversity exploration of marine unicellular eukaryotes [24, 25]. In these studies, single cell genome amplification was combined with ribosomal RNA-based identification and low-coverage shotgun sequencing. These presumed single amplified genomes obtained from the individual protozoan cells, were revealed as reduced-complexity metagenomes, featuring also interacting viruses and bacteria. With regards to *Cryptosporidium*, some preliminary work has been published on the potential use of flow cytometry to detect and quantify *Cryptosporidium* oocysts from water and fecal samples [18, 26, 27], but so far, these efforts have not been extended to full genome characterization. However, in *Cryptosporidium meleagridis* attempts has been made. Keely at al presented preliminary data from a single oocyst at a The IWOP conference in 2012 [28]. However, the data and experimental procedure was never published.

*Cryptosporidium* represent a suitable platform for rigorous and transparent method development and validation to guide future application of single cell genomics methodology to both parasites and other environmental microeukaryotes. To dissect genome-wide variation among individual oocysts within a clinical *Cryptosporidium* infection, we established a workflow for whole genome sequencing of single cells. With validation of assay performance, both with regards to success rate, coverage and precision, the door opens for further understanding of parasite populations within and between infections. The exploration of the hidden diversity of microeukaryotes may begin!

## Results

**Fig. 1:**
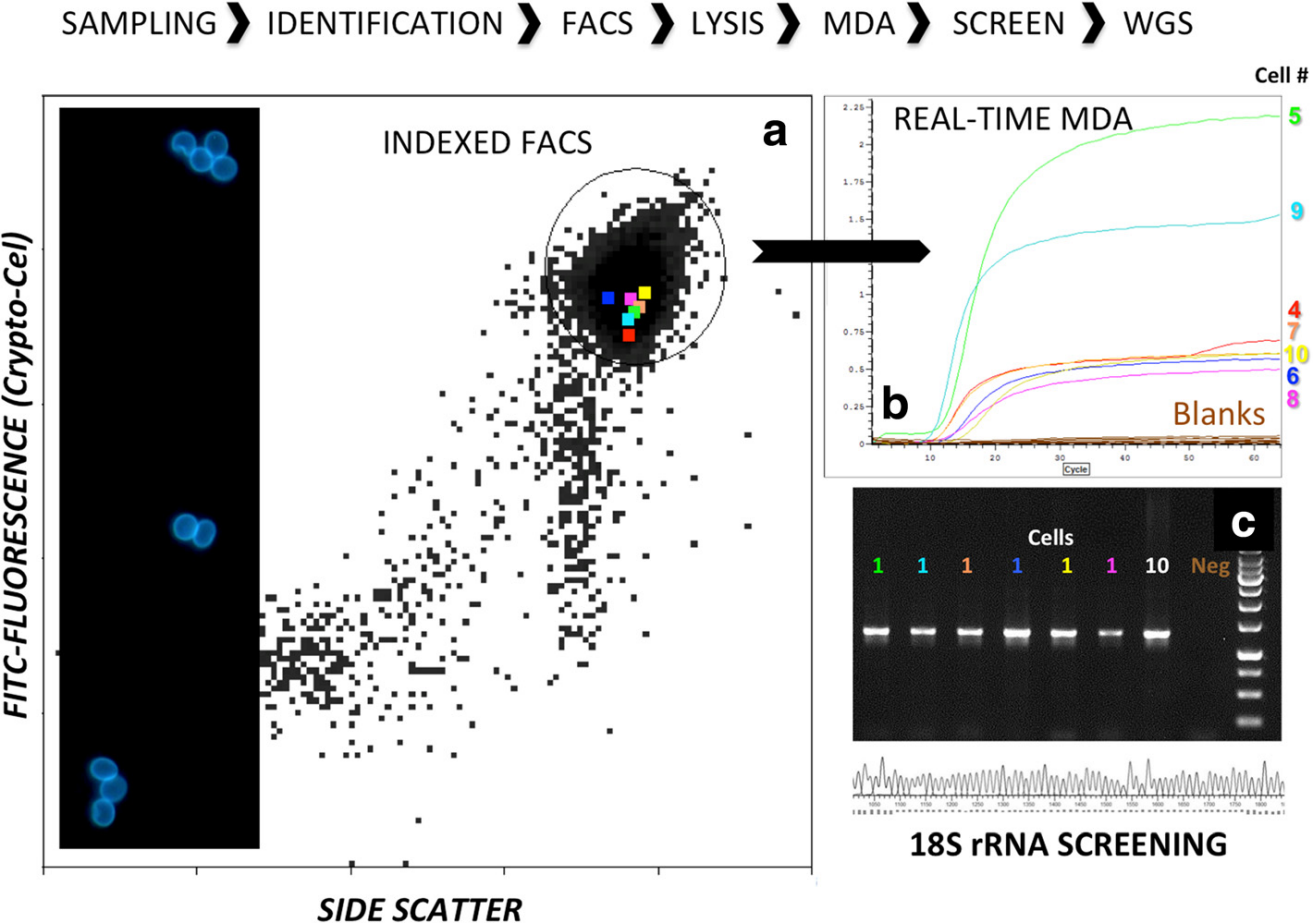
Conceptual graph of single cell genomics workflow applied to *Cryptosporidium parvum*. **a** Scattergram of side scatter and antigen-based fluorescence used for sorting individual cells. The gate for sorting is indicated by a circle and individual analyzed cells are marked by color. Panel with antibody stained oocysts to the left. **b**. Real-time MDA kinetics for individual amplified genomes color coded as in (**a**). Blanks are given in brown. **c** Small subunit rRNAscreening of amplified cells with gel electrophoreses and sequencing. Negative control and positive control with 10 cells are included for comparison

To optimize the single cell genome workflow for protozoan parasites, 50 *Cryptosporidium* sp. cells tagged with fluorescent antibodies were individually FACS sorted (Fig. 1) and subject to alkaline heat lysis and subsequent whole genome amplification. Cells used for this analysis was obtained from a clinical isolate (Uppsala1499) from a calf. The success rate for the combined sorting, lysis and amplification was 80 %. For all of the 40 positive samples (100 %), a single-step PCR screen with genus-specific 18S rRNA primers produced amplicons of the expected size. Sanger sequencing confirmed that the amplified genes matched a previously verified 18S rRNA gene from *Cryptosporidium parvum* IowaII (NCBI accession number AF164102).

**Fig. 2:**
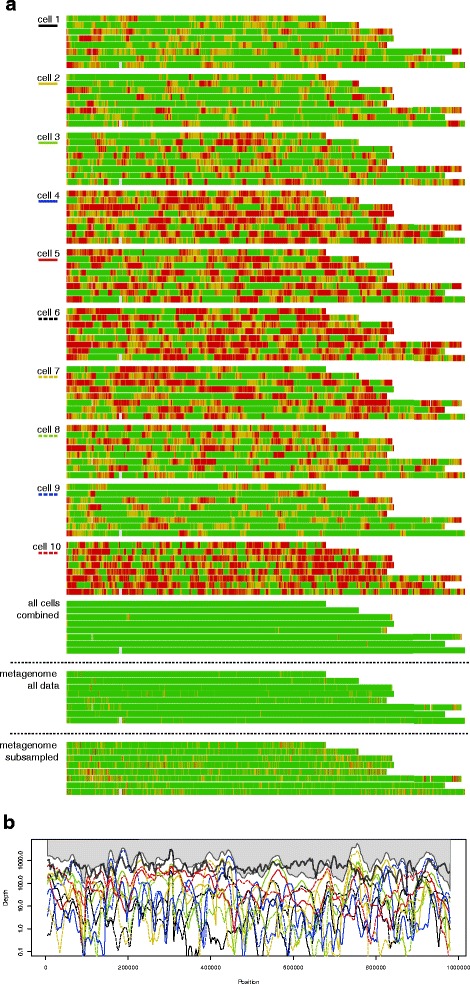
Coverage statistics for single cell genomes of *Cryptosporidium parvum*. **a** Color coded heat map for the eight chromosomes of individual cells where red is < 1 × coverage (sequence missing), orange is 1–5 × coverage, yellow is 5–20 × coverage and green is >20 × coverage. Positions with ambiguity characters in the reference genome are colored gray. The combined data for all 10 single cell genomes (all cells combined), the full parent metagenome and the same metagenome subsampled to equal sequencing depth as an average single cell genome (1.3 Gbp) are included for reference. **b** Density profiles showing more detailed view of the sequencing depth for chromosome 3 with the ten single cell genomes given in color according to (**a**). “All cells combined” are shown with gray shading. Similar plots for all chromosomes can be found in Additional file 4. The bold black line represents the metagenome

**Fig. 3:**
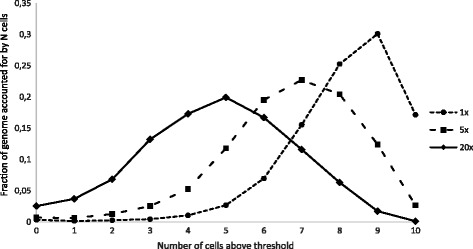
Fractions of genome shared among single amplified genomes (0 to 10 cells) from *Cryptosporidium* sp. The “fraction of genome accounted for by N cells” at 0 “number of cells above threshold” represents the portion of the reference genome not accounted for in any of the cells at the indicated coverage. In contrast, the portion of the genome accounted for in all cells is found at 10 cells on the X-axis. Observed shared fraction of the genome is given at coverage cutoffs of 1, 5 and 20 ×

Illumina MiSeq sequencing of 10 single cell genomes generated a total of 13.4 giga-basepair (Gbp) sequence data. On average 99.8 % of the sequence data mapped to the *Cryptosporidium parvum* IowaII reference genome (Table 1). The *Cryptosporidium* sequences were distributed across the 10 single cell genomes with on average 1.3 Gbp per sample (range 0.3 to 1.9 Gbp). The portion of the reference genome accounted for in the individual single cell genomes was on average 81 % (range 67–95 % for mapped reads). Combining sequence data from all single cell genomes, almost the entire reference genome (99.7 %) was accounted for and most of this (98.8 %) was described at > 20× coverage (Figs. 2 and 3). The isolate (Uppsala1499) was also subjected to direct sequencing for comparison (hereafter referred to as "metagenome"). This sequencing generated 6.3 Gbp sequencing data. In comparison, the metagenome covered 97.8 % of the genome, of which 84.8 % was covered by at least 20×. Subsampling the metagenome to the same sequence volume (total bases) as the average single cell genome, 92.2 % of the genome was covered at least 1× and 62.2 % had a coverage exceeding 20×. It must however be noted that the metagenome was generated with MiSeq v2. (2 × 250 bp) instead of v.3 (2 × 300 bp) which was used for the single cell genomes, possibly biasing this comparison. There was no difference in average GC content between the assembled single cell genomes (average 30.8 %) and the Uppsala1499 genome (30.2 %).

**Table 1:** Genome properties. Reads were either mapped to the *Cryptosporidium parvum* IowaII reference genome or assembled *de novo* using Spades

	Total sequence (Mbp)	Read length (bp)	Mapped reads (%)	Fraction of genome covered (%)	Sequencing coverage (X-fold +/− SD)	De Novo Genome Assembly Statistics			MDA statistics		
						Size (% of reference)	Longest contig (bp)	NG50	Ct (h)^a^	Effic-iency^b^	Yield (AU)^c^
Cell 1	920	2 × 300	99.8 %	94.7 %	90.3 ± 157	91.8 %	225 980	57 459	2.3	70	0.85
Cell 2	1366	2 × 300	99.9 %	93.8 %	127 ± 225	90.7 %	227 055	64 805	3.0	65	0.74
Cell 3	2013	2 × 300	99.8 %	87.8 %	169 ± 540	80.6 %	147 412	20 114	3.0	123	1.69
Cell 4	341	2 × 300	99.6 %	69.4 %	30.6 ± 79.4	60.0 %	67 943	1 513	2.8	99	0.69
Cell 5	1218	2 × 300	99.8 %	74.9 %	105 ± 307	66.3 %	119 204	4 309	3.3	91	0.56
Cell 6	1460	2 × 300	99.9 %	68.4 %	111 ± 509	57.1 %	73 330	1 398	2.3	97	2.17
Cell 7	1470	2 × 300	99.8 %	78.8 %	90.3 ± 304	67.6 %	203 151	5 364	2.8	102	0.60
Cell 8	1460	2 × 300	99.8 %	85.5 %	112 ± 284	77.5 %	117 185	11 229	2.3	192	1.52
Cell 9	1360	2 × 300	99.8 %	94.9 %	118 ± 194	91.6 %	547 655	65 774	3.3	44	0.50
Cell 10	1877	2 × 300	99.8 %	66.9 %	115 ± 767	49.6 %	57 954	687	3.5	72	0.60
Meta-genome	6273	2 × 250	84.2 %	97.8 %	535 ± 829	94.4 %	95 102	7 076	N.A.	N.A.	N.A.

^a^The number of hours to reach above the background fluorescence threshold

^b^The exponential amplification of DNA in the exponential amplification phase where a value of 100 % represents a doubling in DNA concentration every 15 min

^c^The total DNA fluorescence (arbitrary fluorescence units) at the end of the 16 h MD

Inspecting the individual single cell genomes, it was evident that amplification was uneven along the genome. Certain portions of the genomes were covered up to 25,000-fold, whereas other regions from the same single cell genome had no coverage (Fig. 2, Additional file 1). There were no apparent regions that were consistently preferentially amplified across all the single cell genomes. Also the non-amplified metagenome sample feature some less pronounced variation in coverage along the genome (Fig. 2b), hence at least some of the uneven coverage is likely to have been introduced in the library preparation for sequencing or the sequencing itself. A few shorter regions appeared not to amplify at all in any of the samples. Most of these supposedly missing regions were upon closer inspection represented by ambiguous bases in the IowaII reference genome sequence. This would explain why these regions (colored grey in Fig. 2) could neither be mapped for the metagenome, nor the single cell genomes. To illustrate the portion of the genome that can be used for assessment of genetic variation across the single cell genomes, a cumulative nucleotide-by-nucleotide comparison was made (Fig. 3). Despite the uneven amplification and a quite limited sequencing effort, > 95 % of the entire genome was covered in five cells. Robust SNP calling would typically require higher coverage and for the same number of cells, 78 % of all nucleotide positions had at least 5× coverage (Fig. 3).

For some organisms, reference sequences are not available for genome mapping. In such cases, there is a need to perform *de novo* assembly prior to annotation and variation analysis. This could be challenging for genome sequences with uneven coverage. Using a standard *de novo* assembler designed for single amplified genomes (Spades v. 3.6.1), the resulting representation of the reference genome ranged from 50 to 92 % for the individual single amplified genomes. Contigs varied in size up to 548 Kbp and the distribution of contig lengths varied dramatically, as evident from the NG50 which ranged from a mere 687 bp to 66 Kbp between the individual cells (Table 1). It is notable that the large variation in quality is because of one single cell genome with overall very poor performance (Cell 10) whereas three cells were assembled *de novo* at more than 90 % representation of the reference genome and NG50 above 57,000 (Table 1). When matching the assemblies of the different cells to each other, a rate of 10–40 mismatches/100,000 bases was observed, implying a nucleotide-level assembly accuracy of approximately 99.99 %. The NG50 for the de novo assembly of the metagenome was 7076 bp. The quite poor quality of the metagenome was likely caused by damage to the genome caused by prolonged storage of oocysts since the metagenome was sequenced later than the single cells. At the level of individual nucleotide positions, 3560 polymorphic sites (SNPs) were identified relative to the IowaII reference genome when pooling all 10 single cell genomes (Table 2). Most of these (3350) were shared with the metagenome and were also identical across all individual single cell genomes, thus representing inter-population variation. The remaining 210 variable sites were evenly spread across all the sequenced single cell genomes when compared to one another and to the Uppsala1499 metagenome (Additional file 2). All identified SNPs towards the Uppsala1499 were unique to one single cell and ranged from 13 to 51 per cell.

**Table 2:** Genomic variation relative the *Cryptosporidium parvum* IowaII reference genome or the Uppsala1499 metagenome

SNPs	Single cells^a^vs. IowaII	Single cells^a^ vs. Uppsala1499	Uppsala1499 vs. IowaII
Total	3350	210	3874
C > T	545	91	622
G > A	583	105	669
A > G	584	1	679
T > C	559	5	667
T > A	174	1	204
A > T	172	2	197
C > A	151	3	153
G > T	198	0	227
G > C	47	1	58
C > G	57	1	67
T > G	142	0	170
A > C	138	0	161

^a^Statistics refer to the 10 single cell genomes combined

There was an extreme bias among the 210 cell-specific base substitutions towards one kind of transition (C- > T and G- > A) as shown in Table 2. These transitions represented 93 % of all substitutions and the transition vs. transversion ratio was 25.3. In contrast, the 3350 SNPs that were identical in all single cells as well as in the metagenome when mapped to the IowaII sequence, featured a transition vs transversion ratio of 2.1 (Table 2). This bias may indicate that this apparent genetic variation is an artifact from the amplification or sequencing. Accordingly, only one SNP found in a single cell could be verified in the metagenome, where it was present in ~30 % of the population. Hence this single SNP represents true genomic variation within the Uppsala1499 isolate. This transversion at position CM000429:646664 in the cgd1_2980 gene, encoding the 14-3-3 protein, causes a missense substitution of alanine to glutamine.

The *Cryptosporidium* oocysts are composed of four sporozoites, each with a haploid genome. Hence genetic variation within individual oocysts would be seen as variable nucleotides with alternative bases at 25, 50 or 75 % of the reads. No overrepresentation of such base fractions could be detected in the cells (Additional file 3), suggesting that all sporozoites in a single oocyst have the same genetic composition. All 3560 SNPs were further examined for what translational consequences they have. For the 3350 SNPs identified vs. IowaII, the majority were found either outside coding regions or represented a synonymous substitution. Only three were identified as causing stop codons and a third gave rise to missense substitutions. In contrast, for the remaining 210 mutations, most substitutions caused a codon change or introduced a stop codon (Table 3).

**Table 3:** Effect of detected mutations

Comparison # of SNPs	All vs. IowaII (#3350)	Single cells vs. Uppsala1499 (#210)
Downstream	28 (0.8 %)	2 (1.0 %)
Upstream	1066 (31.8 %)	38 (18.1 %)
Synonymous	1063 (31.7 %)	40 (19.0 %)
Missense	1180 (35.2 %)	118 (56.2 %)
Stop gained	3 (0.1 %)	12 (5.7 %)
Start lost	1 (0.0 %)	0 (0.0 %)

The large variation in the quality of the sequenced amplified genomes from the individual *Cryptosporidium* cells (Table 1) calls for robust ways to predict these features and select the amplified genomes of highest quality for downstream full genome sequencing. The single cell genomics workflow used in the present study has several quality control steps and assay performance monitoring parameters that can be used for this purpose. Firstly, the FACS employed enables index sorting, e.g. the fluorescence and scattering properties of individual sorted cells can be recorded and used for selection. The sorting here was based on FITC fluorescence from surface presentation of the marker proteins and side scatter, both of which can provide information on the physiological state of the cell.

Secondly, the progression of the whole genome MDA is traced in real time using intercalating fluorescent dyes for DNA quantification and this amplification can be characterized both by the time required until a certain amplification threshold is reached (Ct), amplification efficiency (slope) and DNA yield (maximum fluorescence). It was evident that none of these variables in isolation could be used for predicting the observed genome coverage (Table 1). Furthermore, even after combining the MDA variables and cellular properties from the FACS in a Partial Least Squares Regression model (PLS), the predictive power of the model was low with a root mean square error (RMSE) of 22.5 %, representing the average difference between modeled and observed genome coverage for the analyzed genomes (Additional file 4). Cellular properties (surface antigen fluorescence and forward scatter as a proxy for cell size) were the factors with the strongest positive correlation to genome coverage, whereas the commonly used Ct value used for identifying successful MDA reactions was negatively correlated to observed genome coverage.

## Discussion

In this study we have benchmarked the use of single cell genomics for genome-wide characterization of individually sorted and free-living microscopic eukaryote cells. From a diagnostic and infection biology perspective, there are several reports on mixed infections (several species as well as several subtypes) within the same host [6, 29–31]. The clinical importance of mixed infections is poorly understood and not well studied, as detection of multiple species/subtypes is elusive with existing typing methods, whereas single cell genome characterization holds great potential in this regard. Furthermore, the available markers only cover a minimal part of the genome and more information could deepen our understanding about genome-wide variation at the population level. More information based on such variation and on additional genes, could also aid in building a better knowledge about the implications of genetic diversity within a single host. This type of knowledge has been shown to be important for other infectious protozoans [32, 33].

More broadly, earlier work has shown that most unicellular eukaryotes in the biosphere have not been successfully cultivated and hence we have incomplete understanding of their evolutionary history and only partial knowledge about their functional attributes [24, 25, 34, 35]. The workflow established and tested here, using *Cryptosporidium* as a single cell genomics testbed, can thus serve as a foundation for future studies expanding to include other taxa and lineages after some methodological adaptations.

*Cryptosporidium* oocysts are dormant and mechanically robust [36]. Accordingly earlier work to isolate genomic DNA from such cells have had mixed success [36, 37]. The lysis and DNA extraction success cannot be easily determined, but as a conservative estimate of the performance, we observed 80 % success rate for the combined sorting, lysis, MDA and 18S rRNA screening for our clinical *C. parvum* sample. Considering that previously reported success rates for these combined steps rarely exceed 40 % for other microorganisms [38], our results are encouraging for future expansion also to other microeukaryotes.

All genome sequences presently available from clinical *Cryptosporidium* isolates have been produced following a step of IMS [3, 10–12]. This method depends on the specificity of the antibodies used. We have observed that for some species, and also in case of isolates within *C. parvum*, the IMS is less effective (data not shown). This is likely to selectively exclude some species or subtypes in the purification process and the true population will not be reflected in the genome sequence (metagenome) nor in the set of single cell genomes. In the present study, we have not used IMS for purification of cells used for single cell genomics and could therefore bypass this problem. However, we did use antibodies for the FACS which will again represent a bottleneck for those oocysts where the antibodies do not bind. This could, in future experiments when a true representation of all variants is critical, be overcome by using other parameters in the cell sorting, such as light scatter and DAPI staining for cell selection. Our results show that the purification protocol used is sufficient to provide clean oocysts without other DNA containing contaminants, and combining sorting based on DNA content and a genus specific PCR after the WGA would ensure a non-selective workflow.

The high genome representation (average 81 % for 10 genomes distributed on a single Illumina MiSeq run) and high precision is encouraging and suggest that the genomic features of successfully sorted unicellular eukaryote cells can be accurately described, as long as DNA is efficiently recovered. The only previous publication on large-scale whole genome sequencing of protists reported an average 62 % representation for *Plasmodium* single amplified genomes [23]. For comparison, whereas recent large-scale single cell genome sequencing of *Prochlorococcus* cells reported an average 70 % representation of this much smaller (1.6 Mbp) cyanobacterial genome [39].

Nair et al. [23] also searched the *Plasmodium* genomes for known SNPs previously identified in the VeraCode assay [40]. This analytical strategy is affordable and fast and would enable complex infections to be resolved and population structure to be described. However, using this targeted methodology for only a limited number of variable sites, there is a high likelihood of overlooking errors introduced during genome amplification and sequencing. Such information would be of critical importance when identifying novel variants of an organism or sequencing completely new microeukaryotes. We took an alternative approach and carried out single cell genome sequencing followed by a genome-wide analysis based on both comparison to reference population genomes and *de novo* assembly. Our work demonstrates, for the first time, that it is possible to obtain high quality genome data from unicellular eukaryotes with regards to both coverage and precision. This holds great promise for future biological discovery and description of unknown genomes with single cell genome sequencing.

It should be emphasized that the reasoning and need for collecting single cell genome data can vary, and sometimes SNP based genotyping will be sufficient (e.g. for straightforward diagnostics involving distantly related populations). However, if the aim is to describe functional properties, the demands on the data are quite different in terms of cells analyzed and genome overlap among these cells. The observed stochastic variation in amplification of different regions of the genome will impair the possibility to compare all part of the genome between any pair of genomes. However, we also demonstrate that with only 10 cells analyzed, approximately three quarters of the individual positions in the genome will be represented in the vast majority of single cell genomes. This offers great potential for carrying out genome wide population genetics studies and describe multiple infections or population divergence in complex environmental samples.

Our data further shows that it is possible to separate true variation from background while it is also clear that his requires careful interpretation. Although robust, phi29 introduces errors at a rate of approximately 1 × 10^−5^ [41, 42]. Therefore, the expected number of errors introduced for a complete first copy of the 9.1 Mbp genome is close to 100. Comparing our single amplified genomes to a metagenome from the same population, the reported number of SNPs varied from 13 to 51. We argue that almost all of these SNPs can be explained by expected early-stage amplification errors where the somewhat lower SNP frequency is caused by the presence of multiple genome copies in each *C. parvum* cell. It is quite straightforward to identify such errors when a well-characterized population genome is available, and this comparison also enabled us to identify a single SNP representing true genome variation. Furthermore, our results clearly show that certain transitions (C > T and G > A) represent the majority of the false diversity caused by early phase phi29 amplification. Accordingly we recommend that observation of these specific transitions should be interpreted cautiously in single cell genomes.

Single cell genomics can be applied in different ways, both in basic science and for clinical applications [16, 23, 33, 38]. For protozoan parasites in general and specifically for *Cryptosporidium* sp., our workflow can be used to perform whole genome population studies targeted at the ultimate biological resolution of a single cell. This can be used to disentangle biological aspects, such as impact of disease, of interactions among co-infectants in a multiple infection, so called poly-parasitism [43]. Using single cell genomics we can, in a population, find rare variants of species or alleles associated with traits such as virulence or resistance. These rare variants would, in normal NGS be lost as assumed sequencing errors. In addition, we demonstrate that *de novo* assembly is tractable at the level of individual single cell genomes and with almost the same success as mapping to a closely related reference genome scaffold (Table 1). This opens the door to reconstruction of entirely new genomes, capabilities that are particularly important in biodiversity research targeting other unicellular eukaryotes where no reference genomes are available. This would likely entail some modification of lysis procedures to efficiently recover DNA for the amplification. However, using procedures very similar to the ones used here, Martinez-Garcia et al. [24] were able to amplify genomes from taxonomically diverse marine phytoplankton and protozoans. Although these cells were not genome-sequenced, they could be identified from sequencing of the small subunit ribosomal RNA gene, demonstrating that at least this portion of the genome was recovered and amplified. There is no reason to believe that precision observed for our *Cryptosporidium* single cell genomes would not apply also to such poorly studied eukaryotes, enabling future biodiversity mapping that is resolved to the cellular level while shedding light on metabolic properties, the transfer of mobile genetic elements within populations, genome rearrangements and population genetics without relying on cultivation.

There is no specific treatment to *Cryptosporidium* sp. [2]. However, in case of infections with other parasites developing resistance to antiparasitic drugs, information on the population genetic setup in a specific infection could be important from a therapeutic perspective. The within-host interactions and treatment will strongly affect the evolution of resistance and multiple infections of malaria parasites have been predicted to spread drug resistance [44, 45]. Knowing the resistance status of the population in the patient may guide medication and treatment strategies. The component variants within an infection could be revealed using single cell genomics [23].

Our data suggest little variation among the studied cells within the population based on comparison with the metagenome. However, in only the few studied cells (10) we identified one SNP that could also be verified in the metagenome. In large outbreak situations, detailed genotyping could be important for source tracking purposes and transmission investigations but also to study the population genetic variability in the parasite. In such outbreaks, detailed genotyping of a relevant proportion, let alone all, infected patients is practically difficult, or even impossible. Using the described single cell workflow, either on samples from sewage plants or pooled fecal samples from multiple patients, could enable investigators to perform relatively fast and detailed molecular characterization of the causative agents causing an outbreak. In addition to identification of species and strain of the infectious agent, information of putative zoonotic potential, multiple species and/or strains may be gained.

Many microbial parasites can replicate sexually as well as asexually [45, 46], resulting in either recombining or clonal populations. While standard WGS may detect sexual replication in an isolate by identification of polymorphic sites, there is no currently established method to assign specific polymorphisms to a certain copy of the genome. Using single cell genomics we can shed some light on questions regarding recombination and sexual replication in polyploid and/or multi-nuclei containing microbes, even in unculturable parasites with complex life cycles, such as many of the apicomplexa. In *Cryptosporidium*, each life cycle encompass sexual as well as asexual replication, and thus, if there is variation within the four sporozoites sequenced in each well, we expect to detect polymorphism as quarter fractions of heterozygosity in some loci [47]. In our study we did not detect any such frequencies of polymorphism, a fact that may reflect an original clonality of the isolate we have sampled, or that our study is relatively small, covering the sequence of only 40 copies of the genome.

## Conclusions

In this work, we show that single cell genomics can be applied to describe the diversity and genetically characterize individual unicellular eukaryote cells. We further show, using a clinical Cryptosporidium sample as a testbed, that the coverage and precision of the methodology was sufficiently high for characterization of population genetic structure. Our whole genome sequencing of individual protozoan cells will certainly soon be followed by many others. As single cell genome sequencing is becoming an established tool and sequencing capacity increases, large-scale experiments, epidemiological surveys and global biodiversity mapping will be tractable also at single cell resolution. In learning more about the limits of single cell genome assay performance, the present study can guide future research efforts and ensure realistic goals and efficient use of resources.

## Methods

### Organisms and clinical samples

The *Cryptosporidium parvum* isolate Uppsala1499 (subtype IIaA16G1R1b) was obtained from a calf as a clinical fecal sample. Initial oocyst concentration was determined after flotation as described below. Flotation liquid (10 μl) was spread and fixed on a Teflon coated glass slide and labeled with a parasite-specific fluorescein isothiocyanate (FITC)-labeled antibody (Crypto-Cel, Cellabs, Australia). The number of OPG feces was determined as the average number of labeled oocysts detected in 10 fields of view and was estimated to 2.5 × 10^6^ OPG.

### Purification

To obtain oocysts suitable for single cell sorting, an initial purification procedure was performed including two consecutive flotations, a chlorination step and a final flotation followed by filtration. A fecal sample (5 g) was used for several parallel flotations performed according to Silverlås et al. [48].

The concentrated oocysts were surface sterilized using an equal amount of 2 % NaHClO_2_ and inverted a few times to mix. Sterilized oocysts were pelleted at 1600 g for 10 min and the supernatant discarded. The pellet was washed three times in sterile water. Finally the oocysts were separated from debris using 8 μm pore size membrane filters (Merck Millipore, USA). To test for the presence of bacterial contaminants, 10 μl oocyst suspension was diluted in 190 μl of sterile water and plated out onto Sabouraud agar, blue agar and bovine blood agar. The agar plates were then incubated for 5 days at 20 °C before visual inspection. Additionally, a second set of blue agar and bovine blood agar plates were incubated at 37 °C and Sabouraud agar plates were incubated at 27 °C. No bacterial growth was observed.

For the population metagenome, separate one gram batches of calf faeces were enriched for *Cryptosporidium* oocysts using saline flotation [48]. Oocysts were further purified, from each sample, using two consecutive rounds of target specific immunomagnetic separation (IMS) [10]. Purity and oocyst yields were examined by analyzing 5 μl of each separation using the Crypto-Cel kit (Cellabs, Australia) and visualized in a fluorescent microscope (Olympus B061, UK). For DNA extraction three batches of purified oocysts were pooled prior to oocyst disruption using 0.5 mm zirconia/silica beads (Biospec, USA) in a FastPrep bead beater (MP Biomedicals, USA). DNA was extracted using the QIAamp DNA mini kit (Qiagen, Germany) according to the manufacturer´s recommendations.

### Cell sorting

Prior to sorting, the purified oocysts were inactivated at 65 °C for 5 min. Cells were diluted in water and stained with a FITC-labeled antibody (Crypto-Cel, Cellabs, Australia) to yield a final 60× dilution in the prepared sample. The sorting was performed with a MoFlo Astrios EQ (Beckman Coulter, CA, USA) cell sorter using a 488 nm laser for excitation, 70 μm nozzle, sheath pressure of 60 psi and 1 % NaCl as sheath fluid. Individual cells were sorted into 96-well plates (Biorad, CA USA) containing 1 μl of 1× TE using the single cell sorting mode, 0.5 drop envelope and sorting regions based on side scatter and FITC fluorescence detected at 513 nm using a 40 nm bandpass filter. Index sorting was used and also included forward scatter (FSC) for collection of fluorescent properties of the individual analyzed cells for prediction of genome sequencing success based on partial least squares regression analysis using SIMCA (Umetrics, Umeå, Sweden). Sorting precision for the applied single cell sorting was determined using 10 μm fluorescent beads. Among 1536 wells with individually deposited beads, less than 1 % contained doublets.

### Whole genome amplification using MDA with phi29

Cells were first lysed by adding 1 μl of buffer D2 alkaline solution prepared as described in the REPLI-g- Mini/Midi manual (Qiagen, Germany) to each of the wells and incubating the plate at 95 °C for 60 s in a thermocycler. Samples were neutralized by adding 1 μl REPLI-g Stop Solution (Qiagen, Germany).

MDA was performed using the RepliPHI™ Phi29 Reagent set (0.1 μg/μl, RH04210, Epicenter, WI USA) at 30 °C for 16 h in 15 μl reaction volumes with a final concentration of 1× reaction buffer, 0,4 mM dNTPs, 10 μM DTT, 5 % DMSO, 50 μM random hexamers with phosphorothioate bonds at the two last nucleotides in the 3´-end (IDT Integrated DNA Technologies, USA Iowa), 40 U Phi 29 enzyme; 0.5 μM SYTO13**®** (Life Technologies, CA USA) and water. All reagents except the SYTO13 DNA stain were twice UV decontaminated at 0.5 J in a Stratalinker. The progression of the MDA (increase in DNA over time) was monitored in real time with SYTO13 fluorescence using a Chromo4 real time PCR instrument (Biorad, CA USA). The reaction was terminated in a heat-inactivation step for 3 min. The amplified DNA was stored at −20 **°**C until further PCR screening, library preparation and Illumina sequencing.

### Screening of amplified genomes

Presence of *Cryptosporidium* in positive genome amplification reactions, defined by amplification curves rising above the background fluorescence within 4 h (Fig. 1), were confirmed by PCR. An aliquot from all wells, both positive and negative, were diluted 40-fold in sterile water and screened using PCR targeting *Cryptosporidium* sp. 18 S rRNA gene. Primers used were 5'- TTC TAG AGC TAA TAC ATG CG-3' and 5'- CCC TAA TCC TTC GAA ACA GGA-3' [49]. The reactions were performed in 20 μl reaction volume with 0.8 U of Taq DNA Polymerase recombinant (Thermo Fisher Scientific, MA USA), 1× reaction buffer, 0.2 mM dNTPs, 3 mM MgCl_2_ and 0.25 μM of each primer. Following a 3 min denaturation at 95 °C, targets were amplified for 35 cycles of 95 °C for 30 s, 50 °C for 30 s, 72 °C for 60 s and a final 10 min extension at 72 °C. Products were detected by EtBr staining and transillumination after electrophoretic size separation on a 1 % agarose gel. Amplicons from seven of the positive PCR products were purified using the NucleoSpin Gel and PCR clean-up purification kit as recommended by the manufacturer (Macherey-Nagel, Germany) and quantified using the Quant-iT ™ PicoGreen® dsDNA assay kit (Invitrogen, MA USA) in a FLUOstar® Omega microplate reader (BMG Labtech, Germany) and submitted for Sanger sequencing identification according to specifications at the Uppsala Genome Center. The sequences were all identical to *Cryptosporidium parvum* Iowa II whilst the remaining three sequences were verified using gel band size of approximate 1300 bp.

### Illumina MiSeq sequencing

Ten single cell genomes (SCGs) were selected based on Ct–values not exceeding 4 h, a clear positive PCR product and, in most cases, an identified Sanger sequence as described recently. The SCGs were quantified with Quant-iT ™ PicoGreen® dsDNA assay kit and diluted to a final concentration of 0.2 ng/μl corresponding to the recommended Nextera XT library input amount. The diluted SCGs were further prepared for NGS sequencing on an Illumina MiSeq instrument using the Nextera XT Library Preparation kit (Illumina, CA USA). Procedures were according to instructions from the manufacturer except that normalization was performed using the Kapa qPCR quantification method instead of bead normalization. In short, the Nextera XT uses an enzymatic step for fragmentation of DNA which enables small quantities of input DNA and includes tagmentation by transposomes linked with adapter sequences. The protocol involves a PCR amplification step where sample-specific Nextera barcodes are also incorporated in the fragments. After PCR cleanup, the libraries for each 10 SCGs were quantified and handed in for individual quality control at the SciLifeLab SNPseq facility. The quality of the libraries was evaluated using the TapeStation from Agilent Technologies with the D1000 ScreenTape. The sequencing libraries were quantified by qPCR using the Library quantification kit for Illumina (KAPA Biosystems, MA USA) on a StepOnePlus instrument (Applied Biosystems, CA USA) pooled in equal amounts prior to cluster generation and sequencing on a single MiSeq run with V3 chemistry and 2 × 300 bp mode.

For the population metagenome, the sequence library was prepared using 1 ng of input DNA and v2 NexteraXT library preparation kit (Illumina) following the manufacturer´s recommendations. After cleanup using AMPure beads (Beckman Coulter, USA), the library was quantified using a Bioanalyzer (Agilent technologies, USA) and diluted to a final concentration of 10pM. The library was sequenced on a single MiSeq run, paired end and 2 × 250 bp mode.

Sequencing data from the 10 cells was mapped to the *C. parvum* IowaII reference genome (GCA_000165345.1) [50] using BWA version 0.7.2. Variant calling was performed with GATK HaplotypeCaller version 3.4-46. Only SNPs were considered, INDELs were ignored. The SNPs were filtered to eliminate potential false positives. SNPs with |ClippingRankSum| > 12.5 or MQRankSum < −12.5 or a FisherStrand value (FS) > 60 were eliminated. Finally, only genotypes with a GenotypeQuality score (GQ) > 20 were considered callable. Only loci where at least four cells were callable were counted in the comparisons. Minority base fractions for all positions in all cells were determined using samtools mpileup.

*De novo* assembly was performed on all samples using SPAdes 3.6.1 with the MDA flag (−−sc) and k values 21, 33, 43, 55, 67, 77, 99, 111 and 127. The quality of the assemblies were evaluated using QUAST [51] with the *C. parvum* IowaII reference genome.

## Additional files

Additional file 1: Density profiles showing detailed view of the sequencing depth for all eight chromosomes with the 10 single cell genomes given in color according to (Fig. 2a). “All cells combined” are shown with gray shading. (PDF 222 kb) Additional file 2: Chromosomal distribution and sequencing depth of the 210 SNPs identified against the parent *Cryptosporidium parvum* metagenome. The eight chromosomes are displayed sequentially on one single axis in numerical order. (PDF 50 kb) Additional file 3: Distribution of base variation for the ten *C. parvum* single cell genomes relative the IowaII reference genome. Each line represents a single cell genome and % reads denote the fraction of the reads with bases that differ from the reference. Positions that, in the SNP analysis, were detected in all cells were excluded from the plot. (PDF 29 kb) Additional file 4: Partial Least Squares Regression model for genome coverage based on MDA and FACS characteristics. A. Weight vector plot for predictor variables (solid squares) and response variables (open square), for the first two latest variables (LV1 + LV2). FL (antibody-derived fluorescence, FSC (forward scatter), SSC (side scatter), Ct (time to reach MDA threshold), Efficiency (efficiency of the MDA) and Yield (final DNA concentration in MDA) is used as predictor variables. B. Correlation plot based on PLS model for observed vs. predicted genome coverage. The root mean square error (RMSE) and the equation for the linear regression is displayed in the graph. Simcas (Umetrics, Umeå, Sweden) was used for the statistical model and all variables were centered at zero and normalized for variance prior to analysis. (PDF 42 kb)
