# Supplementary material for: Cryptosporidium as a testbed for single cell genome characterization of unicellular eukaryotes
Source: BMC Genomics. 2016 Jun 23;17:471. doi: 10.1186/s12864-016-2815-y (PMC4917956; doi:10.1186/s12864-016-2815-y)
Supplement: Additional file 1: — Density profiles showing detailed view of the sequencing depth for all eight chromosomes with the 10 single cell genomes given in color according to (Fig. 2a). “All cells combined” are shown with gray shading. (PDF 222 kb) [file 12864_2016_2815_MOESM1_ESM.pdf]

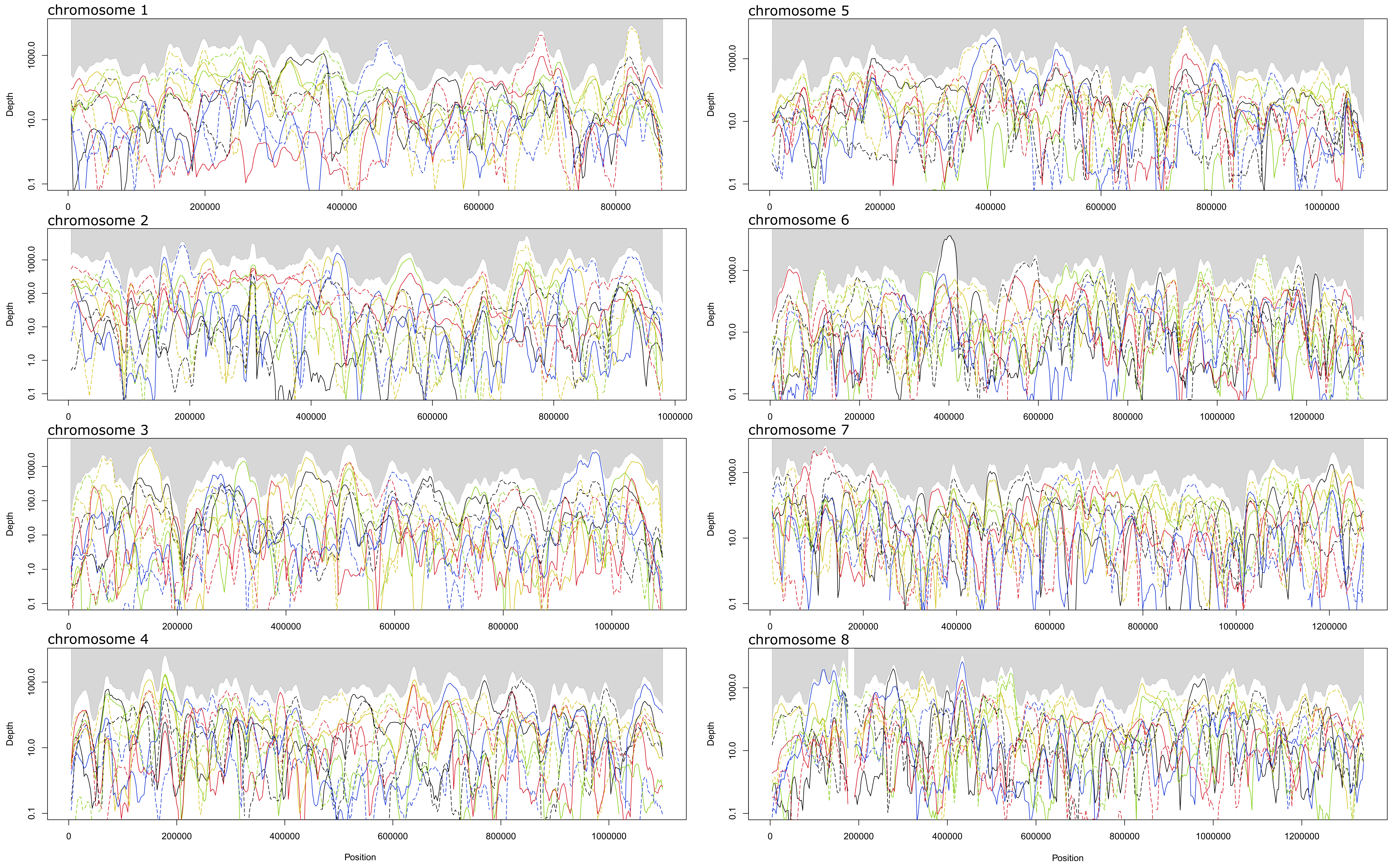

chromosome 1
chromosome 2
chromosome 3
chromosome 4
chromosome 5
chromosome 6
chromosome 7
chromosome 8
Depth
Position
