# Supplementary figures and images for: Cryptosporidium as a testbed for single cell genome characterization of unicellular eukaryotes
Source: BMC Genomics. 2016 Jun 23;17:471. doi: 10.1186/s12864-016-2815-y (PMC4917956; doi:10.1186/s12864-016-2815-y)

**A**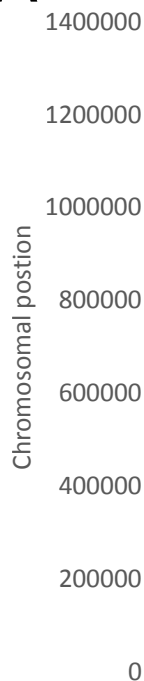**B**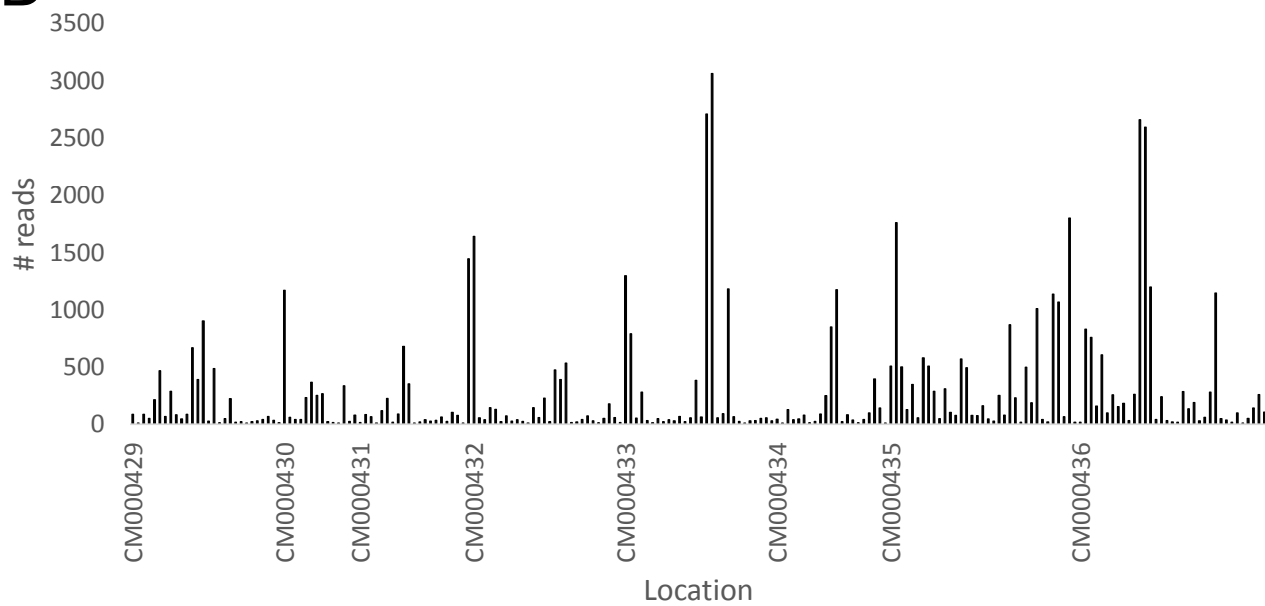

Supplement: Additional file 2: — Chromosomal distribution and sequencing depth of the 210 SNPs identified against the parent Cryptosporidium parvum metagenome. The eight chromosomes are displayed sequentially on one single axis in numerical order. (PDF 50 kb) [file 12864_2016_2815_MOESM2_ESM.pdf]

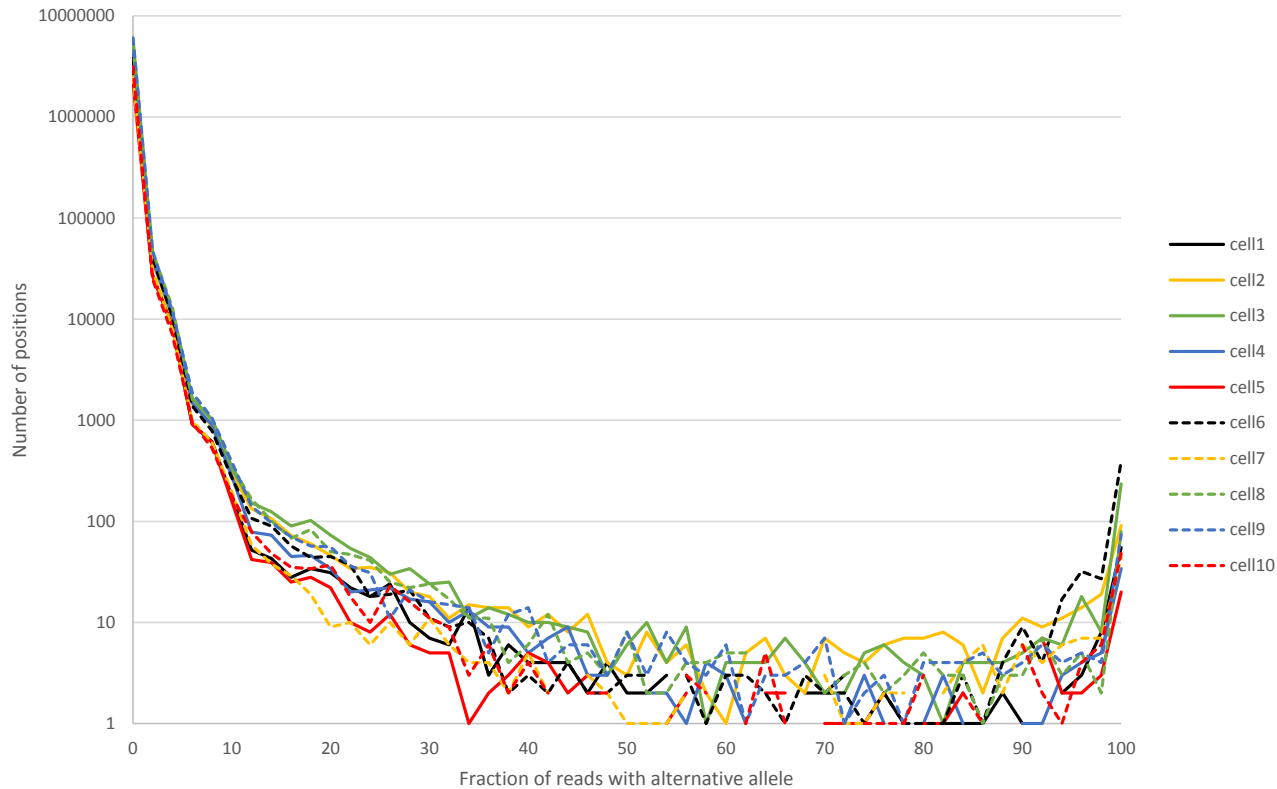

Supplement: Additional file 3: — Distribution of base variation for the ten C. parvum single cell genomes relative the IowaII reference genome. Each line represents a single cell genome and % reads denote the fraction of the reads with bases that differ from the reference. Positions that, in the SNP analysis, were detected in all cells were excluded from the plot. (PDF 29 kb) [file 12864_2016_2815_MOESM3_ESM.pdf]

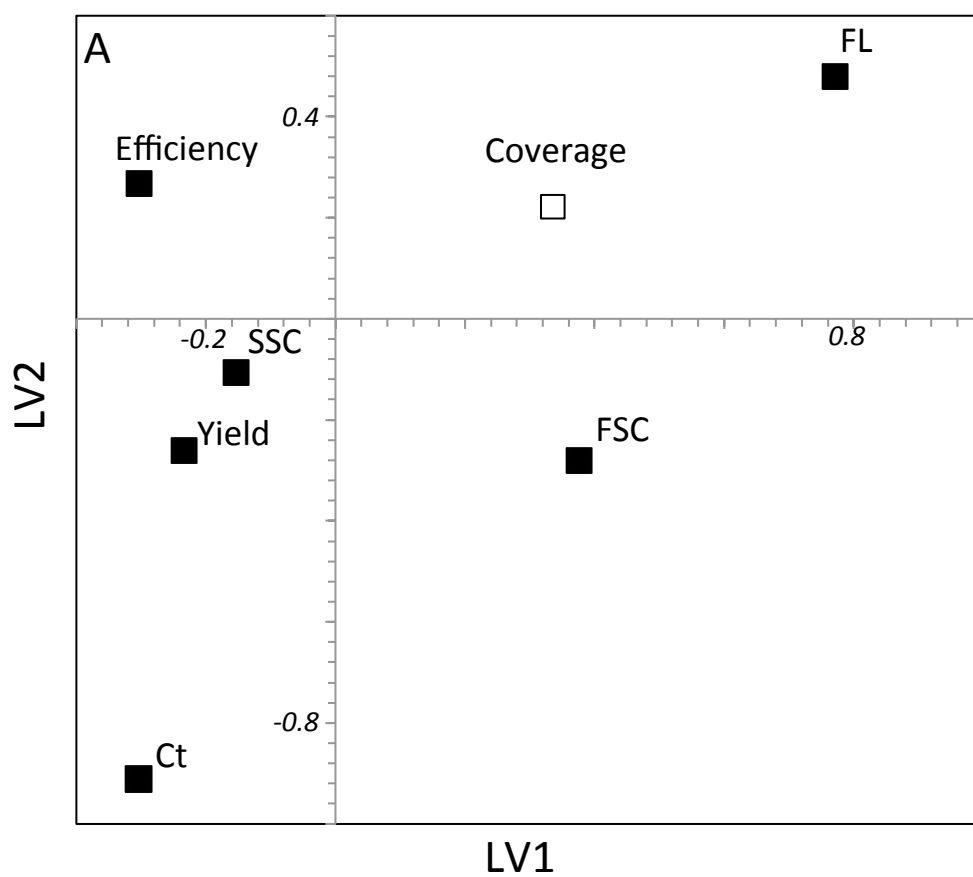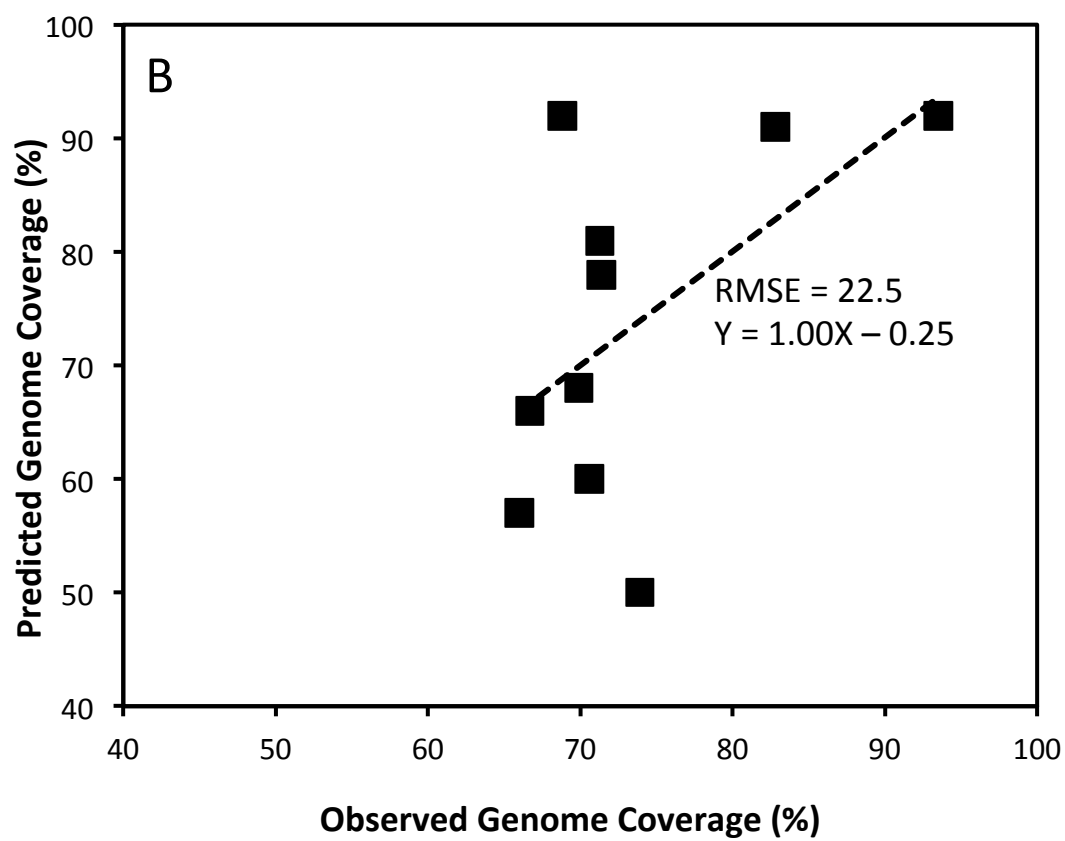

Supplement: Additional file 4: — Partial Least Squares Regression model for genome coverage based on MDA and FACS characteristics. A. Weight vector plot for predictor variables (solid squares) and response variables (open square), for the first two latest variables (LV1 + LV2). FL (antibody-derived fluorescence, FSC (forward scatter), SSC (side scatter), Ct (time to reach MDA threshold), Efficiency (efficiency of the MDA) and Yield (final DNA concentration in MDA) is used as predictor variables. B. Correlation plot based on PLS model for observed vs. predicted genome coverage. The root mean square error (RMSE) and the equation for the linear regression is displayed in the graph. Simcas (Umetrics, Umeå, Sweden) was used for the statistical model and all variables were centered at zero and normalized for variance prior to analysis. (PDF 42 kb) [file 12864_2016_2815_MOESM4_ESM.pdf]
